# The complete mitochondrial genome of the common pandora *Pagellus erythrinus* (Perciformes: Sparidae)

**DOI:** 10.1080/23802359.2018.1467235

**Published:** 2018-05-17

**Authors:** Marina Ceruso, Celestina Mascolo, Elijah K. Lowe, Giuseppe Palma, Aniello Anastasio, Paolo Sordino, Tiziana Pepe

**Affiliations:** a Department of Veterinary Medicine and Animal Production, University ‘Federico II’, Naples, Italy;; b Biology and Evolution of Marine Organisms, Stazione Zoologica Anton Dohrn, Naples, Italy;; c Assoittica Italia, Rome, Italy

**Keywords:** *Pagellus erythrinus*, mitogenomics, Perciformes, Sparidae

## Abstract

The common pandora (*Pagellus erythrinus,* Linnaeus 1758), one of the most popular sea bream species in the Mediterranean Sea, has high potential for aquaculture development. In this investigation, we analyzed the complete mitochondrial genome of *P. erythrinus*. The sequence has 16,828 bp in length and consists of 13 protein-coding genes, 2 rRNA genes, 22 tRNA genes, and a two non-coding regions (D-loop and L-origin). The overall nucleotide composition is: 27.5% of A, 28.2% of C, 27.5% of T, and 16.8% of G.

The common pandora (*Pagellus erythrinus*) is a benthopelagic sparid that is distributed in the eastern Atlantic Ocean and Mediterranean Sea (Sanches 1991). It is also rarely recorded in Scandinavia and in the Black Sea (Bauchot and Hureau 1986). *Pagellus erythrinus* is one of the sparid fishes that are harvested commercially in the greatest amounts in the Mediterranean Sea, and its potential as a significant aquaculture species is recognized (Basurco et al. 2011). However, the genetic information about this species are yet to be adequately addressed. Here, we report the complete mitochondrial genome (mitogenome) of *P. erythrinus* (GenBank MG653592). A specimen caught in the Mediterranean Sea (N 40°22'54.0" E 14°35'44.0") was identified on anatomical and morphological features. DNA extracted from dorsal fin tissue is currently stored at Department of Veterinary Medicine and Animal Production, University ‘Federico II,’ Naples, Italy. The complete mitogenome of *P. erythrinus* was determined by using a combination of long and short PCR, followed by Sanger and Illumina HiSeq 2500 System (Illumina, San Diego, CA) sequencing methods. The complete sequence is 16,828 bp long, containing 13 protein-coding genes, 2 ribosomal RNA genes (12S rRNA and 16S rRNA), 22 transfer RNA genes (tRNA), and two non-coding regions (D-loop and L-origin). Mitochondrial structure and gene organization are in agreement with the typical vertebrate mitogenome (Pereira 2000). The majority of mitochondrial genes were encoded on the heavy strand, with the NADH dehydrogenase subunit 6 (*ND6*) and eight tRNA genes [Gln, Ala, Asn, Cys, Tyr, Ser(UCN), Glu, Pro] being encoded on the light strand. Base composition is 27.5% of A, 28.2% of C, 27.5% of T, and 16.8% of G, similar to other Sparidae mitochondrial genomes (Ceruso et al. 2018). All protein-coding genes started with an ATG start codon with the exception of *COI* and *ND4*, which started with GTG. Four types of stop codon were detected, i.e. TAA (*ND1*, *ND2*, *ATP8*, *ATP6*, *COIII*, *ND4L*, *ND6*), AGG (*COI*), T (*COII*, *ND4*, *CYTB*), and TAG (*ND3, ND5*). The 12S and 16S rRNA genes were located between the tRNA^Phe^ (GAA) and tRNA^Leu^ (TAA) genes, and were separated by the tRNA^Val^ gene as in other vertebrates (Mascolo et al. 2018). The 22 tRNA genes vary from 66 to 74 bp in length. The 1154 bp long control region is located between tRNA^Pro^ (TGG) and tRNA^Phe^ (GAA). The non-coding region (L-strand origin of replication) is 31 bp long and is located between tRNA^Asn^ (GTT) and tRNA^Cys^ (GCA). The phylogenetic position of *P. erythrinus* was examined based on 11 entire sparid mitogenome sequences available in GenBank, using MEGA6 software (Tamura et al. 2013). The species *Lutjanus peru*, *Lutjanus rivulatus*, *Lethrinus obsoletus*, *Chaetodontoplus septentrionalis*, and *Chaetodon auripes* were used as out-group for tree rooting (Figure 1). The resultant phylogeny shows that *P. erythrinus* is closely related to *Dentex tumifrons* and *Pagrus* spp., in agreement with the work of Chiba et al. (2009). The complete mitochondrial genome of *P. erythrinus* will increase our understanding of phylogeny, evolution, and species assignment of sparids.

**Figure 1.**
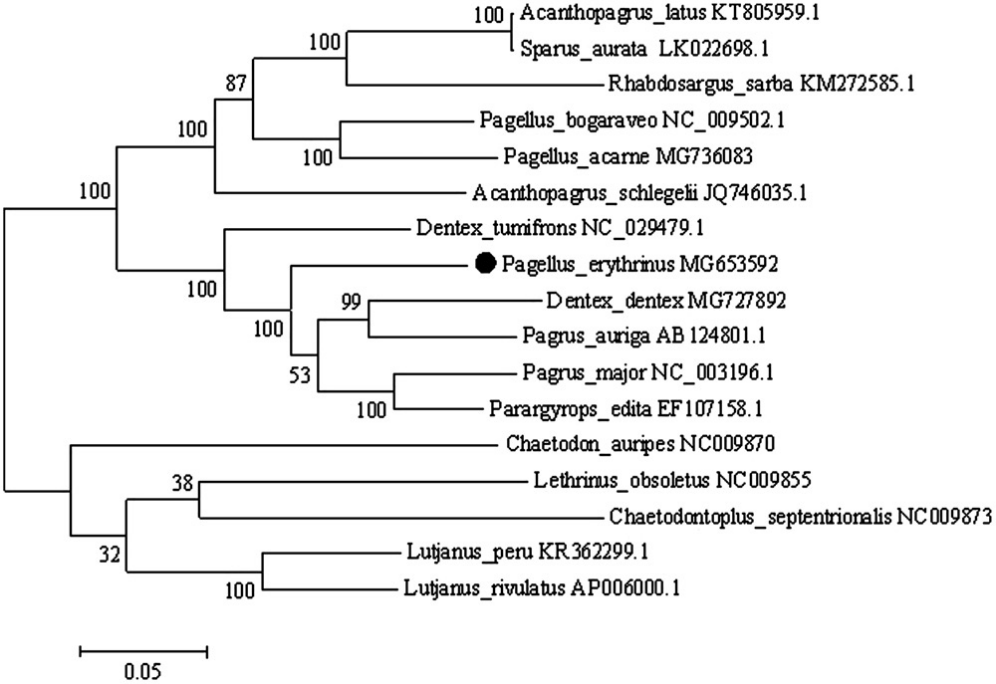
Phylogenetic analysis of *P. erythrinus* based on the entire mtDNA genome sequences of 11 sparids and 5 out-group species by maximum likelihood method. Numbers above the nodes indicate 1000 bootstrap values. Accession numbers are shown behind species names.
